# Automated approach for fetal and maternal health management using light gradient boosting model with SHAP explainable AI

**DOI:** 10.3389/fpubh.2024.1462693

**Published:** 2024-12-20

**Authors:** Nisreen Innab, Shtwai Alsubai, Ebtisam Abdullah Alabdulqader, Aisha Ahmed Alarfaj, Muhammad Umer, Silvia Trelova, Imran Ashraf

**Affiliations:** ^1^Department of Computer Science and Information Systems, College of Applied Sciences, AlMaarefa University, Diriyah, Riyadh, Saudi Arabia; ^2^Department of Computer Science, College of Computer Engineering and Sciences, Prince Sattam Bin Abdulaziz University, Al-Kharj, Saudi Arabia; ^3^Department of Information Technology, College of Computer and Information Sciences, King Saud University, Riyadh, Saudi Arabia; ^4^Department of Information Systems, College of Computer and Information Sciences, Princess Nourah Bint Abdulrahman University, Riyadh, Saudi Arabia; ^5^Department of Computer Science and Information Technology, The Islamia University of Bahawalpur, Bahawalpur, Pakistan; ^6^Faculty of Management, Comenius University Bratislava, Bratislava, Slovakia; ^7^Department of Information and Communication Engineering, Yeungnam University, Gyeongsan, Republic of Korea

**Keywords:** public health, risk perceptions, healthcare, mother and child care, machine learning

## Abstract

Fetal health holds paramount importance in prenatal care and obstetrics, as it directly impacts the wellbeing of mother and fetus. Monitoring fetal health through pregnancy is crucial for identifying and addressing potential risks and complications that may arise. Early detection of abnormalities and deviations in fetal health can facilitate timely interventions to mitigate risks and improve outcomes for the mother and fetus. Monitoring fetal health also provides valuable insights into the effectiveness of prenatal interventions and treatments. For fetal health classification, this research work makes use of cardiotocography (CTG) data containing 21 features including fetal growth, development, and physiological parameters such as heart rate and movement patterns with three target classes “normal,” “suspect,” and “pathological.” The proposed methodology makes use of data upsampled using the synthetic minority oversampling technique (SMOTE) to handle the class imbalance problem that is very crucial in medical diagnosing with a light gradient boosting machine. The results show that the proposed model gives 0.9989 accuracy, 0.9988 area under the curve, 0.9832 recall, 0.9834 precision, 0.9832 F1 score, 0.9748 Kappa score, and 0.9749 Matthews correlation coefficient value on the test dataset. The performance of the proposed model is compared with other machine learning models to show the dominance of the proposed model. The proposed model's significance is further evaluated using 10-fold cross-validation and comparing the proposed model with other state-of-the-art models.

## 1 Introduction

The perinatal mortality rate refers t[o the total number of stillbirths and deaths occurring within the first seven days of life per 1,000 live births. The United Nations International Children's Emergency Fund (UNICEF) data from 2018 indicates that in third-world countries, the perinatal mortality rate is 19, while in developed countries this rate is between 3 and 7 for every 1,000 births, respectively. Sub-Saharan Africa and South Asia are amongst the regions with the highest perinatal mortality reaching 28 and 26 respectively, as per UNICEF (1). At the start of the 20th century, perinatal mortality rates were alarmingly high in the first-world countries of that era, but significant reductions were achieved through improved antenatal care, comprehensive C-section indicators, and perinatal screening technologies like amniocentesis, fetal echocardiography (ECG), amnioscopy, cardiotocograph (CTG), and ultrasound (2). Preterm births, maternal hypertension, birth asphyxia, and septicemia are the main contributors to perinatal deaths and complications related to childbirths (3). Asphyxia is caused by an extended period of oxygen deprivation brought about by interrupted placental blood flow resulting from umbilical cord prolapse, placental abruption, or maternal pre-eclampsia. Asphyxia signs are oversighted lead by delivery mismanagement. Irretrievable organ damage or even death can be prevented by prompt detection of asphyxia signs by fetal heart rate monitoring during the antra and antepartum periods.

Fetal distress presents as an irregularity in fetal heart rate (FHR), either low or high, measured in beats per minute (BPM). Fetal status can primarily be detected through CTG, commonly utilized in clinical examinations (4). Uterine contractions (UC) and FHR are the vital physiological parameters monitored during CTG prenatal checks. Distress can manifest as abnormal FHR, aiding in the early identification of pathological conditions. CTG data enables the classification of fetal health relative to normal parameters. This diagnostic method, conducted during the third trimester of labor, involves continuous monitoring of UC and FHR via pressure transducers and ultrasound probes placed on the maternal abdomen. Real-time readings allow for immediate observation. Clinicians interpret CTG results based on predefined criteria, classifying them as Suspect, Normal, or Pathologic. In first-world countries, CTG stands as a prevalent method for evaluating fetal wellbeing (5). However, some experts oppose excessive CTG utilization in low-risk cases. CTG readings, morbidity, and perinatal mortality are correlated. Neonatal ICUs and low APGAR scores are related to pathological CTG results (6). Fetal distress can also be observed through CTG, and its outcomes vary depending on factors like causative factors, severity, and timeliness of medical interventions. Temporary fetal distress is addressed by changing the position of the mother, injecting IV fluids, administering oxygen supply, or undergoing cesarean sections if necessary, typically toward the end of the third trimester. These interventions aim to enhance the baby's condition and achieve favorable outcomes. However, prolonged fetal distress may result in enduring negative consequences such as intellectual deficiencies, motor impairments, learning disabilities, and disorders like cerebral palsy, albeit rarely (7). Prolonged distress, typically due to oxygen deprivation, may even lead to birth asphyxia, contributing to roughly around 0.9 million neonatal casualties per annum (8).

Fetal death rates are notably higher in low-income countries compared to high-income nations, reflecting disparities in healthcare accessibility and resources between these regions. Despite a global decline in neonatal mortality rates from 36.7 per 1,000 live births in 1990 to 17 in 2020 over the past three decades, the rates remain disproportionately elevated in low-income areas (9). Even in high-income regions, complications of the placenta, often associated with fetal distress, stand as the leading cause of fetal death. Therefore, accurate assessment of fetal health is critical. CTG is an effective source to evaluate and find fetal distress at an early stage. CTG tests offer resource-efficient and timely evaluations, helping ease patient anxiety, particularly in high-volume settings. Certain CTG finding arrangements, such as loss of FHR variability, fixed FHR baselines, and absence of accelerations, signal a non-encouraging fetal status (10, 11). While CTG interpretation traditionally relies on expert visual analysis, automated mechanisms are being explored to augment this process.

Artificial intelligence (AI) has emerged as a valuable tool for assessing the status of the fetus (12). Within healthcare, machine learning is revolutionizing numerous facets, from tailoring treatments to enhancing diagnostic capabilities (13). Progress in health informatics and machine learning algorithms, a subset of AI, facilitates modeling processes, leading to more informed and optimized health decisions (14, 15). Ongoing advancements in machine learning continuously explore practical applications within real-world clinical settings. These algorithms find utility in decision-support systems across various biomedical domains, including fetal classification, aiming to identify compromised fetal statuses and prevent hypoxic injuries and pregnancy-related complications (16). This study proposes a fetal health arrangement framework utilizing a CTG dataset and machine learning. The primary contributions of this research are outlined as follows:

- The study introduces a comprehensive framework for fetal health classification using a light gradient boosting machine (LGBM) and cardiotocogram data.
- The proposed approach uses synthetic minority oversampling technique (SMOTE) as preprocessing for data resampling with the LGBM model for obtaining high accuracy, the area under the curve (AUC), recall, precision, F1, Kappa, and Matthew's correlation coefficient (MCC). In addition, computational complexity is greatly improved.
- The proposed framework is compared with several other machine learning models catboost classifier (CB), extra trees classifier (ET), extreme gradient boosting (CGB), random forest classifier (RF), gradient boosting classifier (XGBoost), decision tree classifier (DT), K neighbors classifier (KNN), AdaBoost classifier (Ada), logistic regression (LR), linear kernel support vector machine (SVM), and Naive Bayes (NB) to show the efficacy of the proposed model. Furthermore, k-fold cross-validation is applied to validate its performance, in addition to, comparing its performance with state-of-the-art models.

The paper is structured as follows: Section 2 presents a comprehensive literature review concerning fetal health classification. Section 3 outlines the proposed framework, including details of the dataset, preprocessing, and the architecture of the proposed classification model, including hyperparameters. Section 4 covers the results obtained and discussions. The discussion is related to the findings and implications of the work. Finally, Section 5 concludes the paper.

## 2 Related works

Multiple research works investigated the application of machine learning in detecting fetal health status. This section delves into the theories and relevant concepts present in current literature, along with their findings, to pinpoint gaps. However, few studies have specifically addressed CTG data-based fetal health classification. Yin and Bingi (17) introduced a machine learning-based system employing XGB, LGBM, and SVM models. The study demonstrated SVM achieving an accuracy of 99.59% for classification. They also compared their system's results with local interpretable model agnostic explanations (LIME) and Shapley additive explanations (SHAP). Abiyev et al. (18) proposed a type 2 Fuzzy neural network (T2-FNN) for discovering the health status of the fetus. They conducted performance comparisons using various machine learning and deep learning models. Implementing T2-FNN with different rule sets, such as 21, 42, and 63 rules, they found that T2-FNN with 63 rules achieved an accuracy of 96.66%.

For early prognosis and classification of fetal health, Kuzu and Santur (19) introduced an ensemble learning model. They utilized ensemble learning techniques including LR, RF, GB, and XGB. The study results indicate that the XGB ensemble learning model surpassed others, achieving CTG dataset-based 99% accuracy. Hussain et al. (20) proposed an improved deep neural algorithm for classifying suspicious CTG recordings and untapped pathological with preferred time complexity. Their system integrates AlexNet architecture with SVM to reduce time complexity at fully connected layers. When a deep transfer learning process is employed, pre-learned topographies are relocated to their prototype. A strategy was implemented by them to further shrink time complexity where other layers were kept in the frozen state while partially training convolutional base layers. The proposed algorithm demonstrated superior performance compared to leading architectures, with instantaneous sensitivities, accuracy, and specificity reaching 96.67%, 99.72%, and 99.6%, respectively.

Piri and Mohapatra (21) proposed a rule-based approach for cardiotocographic fetal evaluation. The work involved utilizing an associative classifier in conjunction with traditional machine learning models. The study highlights that 83% accuracy was achieved by associative classifier prototype before feature selection and 84% after feature selection for classifying fetal health status. Duhayyim et al. (22) introduced an ensemble learning model for automatic fetal health classification. The authors conducted experiments in two scenarios. Initially, they employed five classifiers CatBoost, RF, LGBM, XGBoost, and Ada without oversampling, to categorize CTG readings into pathological, suspected, and healthy. Subsequently, they utilized oversampling deployed ensemble classifiers. They employed random oversampling to balance CTG records for training the ensemble models. Results indicate that XGBoost, LGBM, and CatBoost classifiers achieved 99% accuracy.

Islam et al. (23) conducted research on classifying fetal states using machine learning algorithms. The study employed algorithms such as Ada, KNN, RF, SVM, GBC, DT, and LR. To ensure an unbiased dataset, scaling techniques were employed. Results indicate that GBC outperformed other models, achieving an accuracy of 95%. The study (24) focused on the application of classification techniques in gynecology and obstetrics for CTG data classification to predict normal, suspect, and pathologic cases. Six well-known classification algorithms were evaluated for fetal state classification in CTG datasets. The study revealed that NB achieved a classification accuracy of 83.06%.

Salini et al. (25) conducted a machine learning-based study, addressing the prominence of radical fetal health classification. They deployed various models such as RF, LR, DT, SVC, VC, and KNN on the dataset. The effectiveness of the model was evaluated in classifying fetal health by strenuous training and testing of these models. An outstanding accuracy of 93% was exhibited by the RF model as a promising outcome of this study. Sudharson et al. (26) utilized multiple classification algorithms on the CTG dataset and compared the results of individual algorithms. The comparison involved four different classification algorithms KNN, NB, DT, and SVM. Based on the overall comparison using these classification reports, DT exhibited the highest score among the four algorithms and achieved an accuracy of 90.8%.

Various machine learning techniques, classifiers, and ensemble learning methods have shown promising results in classifying fetal status accurately using the CTG dataset, as evidenced by early research. However, there remains a gap in evaluating these approaches comprehensively, as previous studies have primarily focused on recall, accuracy, F1 score, and precision-like metrics. Additional evaluation parameters such as MCC, Kappa, and training time are necessary to provide a comprehensive understanding of the performance of these methods. A summary of the literature discussed above is presented in Table 1.

**Table 1:** Related work summary.

**References**	**Classifier**	**Performance**	**Limitations**
Yin and Bingi (17 )	XGB, SVM	99.59% SVM	No cross-validation
Abiyev et al. (18)	LR, GNB, SVC, RBF SVC, ANN, CART, RF, RNN, CatBoost, T2-FNN (21 rules,42 rules, 63 rules)	96.66% T2-FNN (63 rules)	No cross-validation, Utilization of imbalanced dataset, and no Explainable AI
Kuzu and Santur (19)	LR, RF, GB, XGB	99% XGB	No cross-validation, Utilization of imbalanced dataset, and no Explainable AI
Muhammad Hussain et al. (20)	RNN, RF, GoogleNet, DesnseNet, NiftyNet, AlexNet, AlexNet-SVM	99.72% AlexNet-SVM,	No cross-validation, Utilization of imbalanced dataset, and no Explainable AI
Piri and Mohapatra (21)	SVM, CBA, DT, KNN, LR, RF, XGBoost, GNB	94% XGBoost and RF	No cross-validation, Utilization of imbalanced dataset, just accuracy is evaluated, and no Explainable AI
Al Duhayyim et al. (22)	RF, XGB, ADA, CatBoost, LGBM, VC	99% XGB, CatBoost, LGBM, VC	No cross-validation, Utilization of less number of features, and no Explainable AI
Islam et al. (23)	ADA, KNN, RF, SVM, GBC, DT, and LR	95% GBC	No cross-validation, Utilization of imbalanced dataset, and no Explainable AI
Afridi et al. (24)	J48, IBK, LR, NB, SMO and RF	83.06% NB	No cross-validation, and no Explainable AI
Salini et al. (25)	RF, LR, DT, SVC, VC, kNN	93% RF	No cross-validation, Utilization of imbalanced dataset, and no Explainable AI
Sudharson et al. (26)	KNN, Naive Bayes, DT, SVM	90.8% DT	No cross-validation, Utilization of imbalanced dataset, and no Explainable AI

## 3 Materials and methods

Fetal wellbeing serves as a crucial indicator for confirming fetal health. Inadequate fetal movement stands as a significant contributor to fetal mortality, underscoring the importance of early diagnosis to promote fetal health. This segment of the research encompasses the following components: dataset description, data visualization, data preprocessing techniques, study deployed ML models, evaluation parameters, and the proposed system for fetal health assessment.

### 3.1 Dataset

We used the Fetal Health classification dataset extracted from the Kaggle repository. This dataset is based on CTG readings of expecting mothers with gestational ages between 29 and 42 weeks comprising 2126 records (27). The author programmed an automated analysis of CTG readings. Signals from Hewlett-Packard fetal monitors and Sonicaid were collected using the SisPorto 2.0 system, comprising 6,000 tested pregnancy records.^1^ Baseline acceleration, fetal heart, contraction, deceleration, and variability readings were established through the input of monitor readings from three experts. Therefore, the obtained dataset forms part of a study to validate the SisPorto 2.0 system. It consists of the individual record-corresponding label with 21 decimal value features, classifying the record into “Healthy,” “Suspected,” or “Pathological” fetal health status.

### 3.2 Data visualization

**Figure 1:**
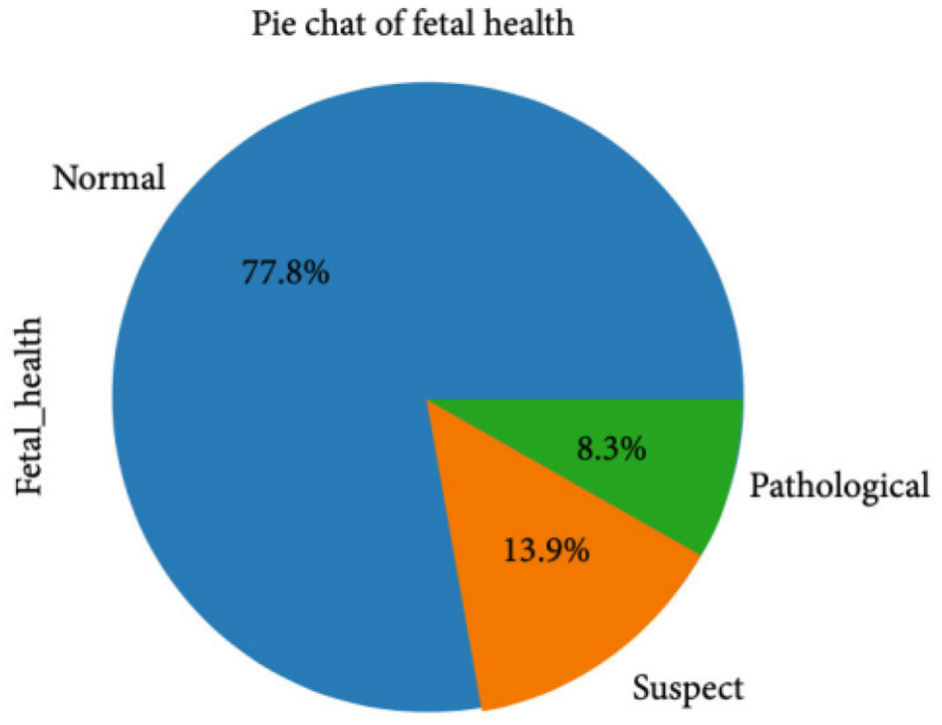
Class-wise dataset representation.

The CTG dataset contains a total of 2,126 records concerning fetal health conditions. Further analysis indicates that the dataset has a class imbalance problem and the distribution of the class samples is not balanced, as illustrated in Figure 1. The dataset has three classes: “pathological,” “normal,” and “suspect,” and has 176, 1,655, and 295 samples, respectively.

**Figure 2:**
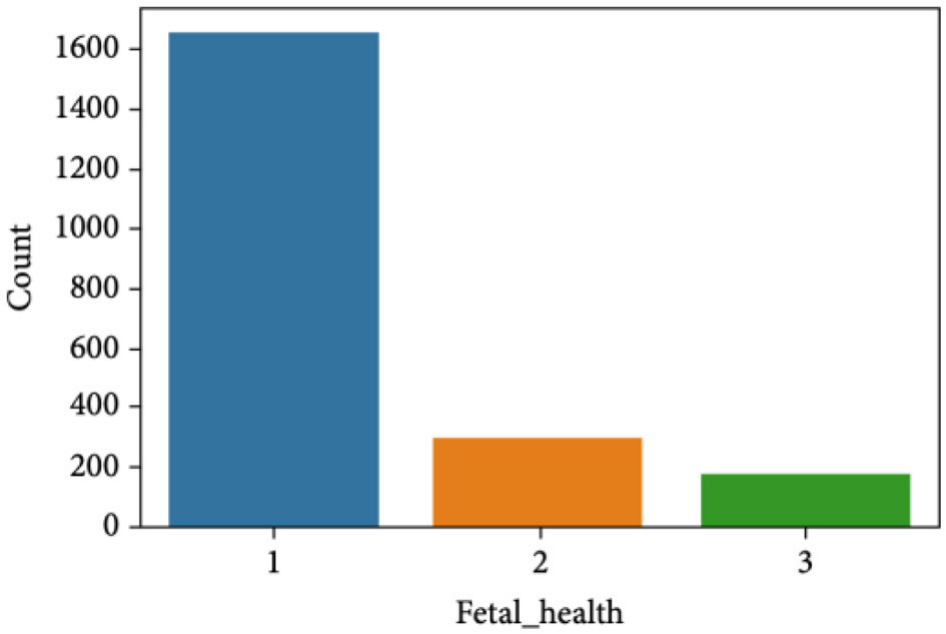
Class-wise dataset distribution before applying SMOTE data upsampling.

Figure 2 clearly indicates the necessity for balancing the dataset due to its inherent imbalance. To address this issue, various techniques were employed to balance the dataset, resulting in 295 suspicious attributes, 1,655 normal attributes, and 176 pathological attributes.

### 3.3 Data resampling

The CTG dataset encompasses readings from labor and random gestation cases. Critical undeniable findings from CTG readings were established despite the low probability of abnormality in these readings. However, the utilized Fetal Health Assessment dataset has class imbalance with a higher ratio of healthy fetuses 1,655 as compared to 176 pathological and 295 suspected occurrences (28). Results can be misleading based on such imbalanced data when used for training a prototype because there is a high probability of the model's learning from a very high ratio of healthy instances in contrast to suspected or pathological cases. Therefore, it's imperative to balance the dataset before constructing a prediction model. Various techniques have been proposed by researchers to address imbalanced datasets, including random under-sampling, random over-sampling, SMOTE, and imbalanced resampling, among others.

In this study, we opted for the SMOTE technique to balance our dataset. SMOTE augments the number of data instances by creating synthetic data points for the minority class using Euclidean distance based on their nearest neighbors (29). These fresh instances being based on the original features look like the original data. While SMOTE may introduce additional noise, it remains a suitable choice for our dataset. Over-sampling with replacement, in particular, serves as a straightforward technique for balancing the dataset, and it aligns well with our dataset characteristics since the CTG records exhibit relatively low variation within a class. Additionally, data integrity is preserved by over-sampling, and the large dataset is facilitated in the process, thereby enhancing model simplification.

**Figure 3:**
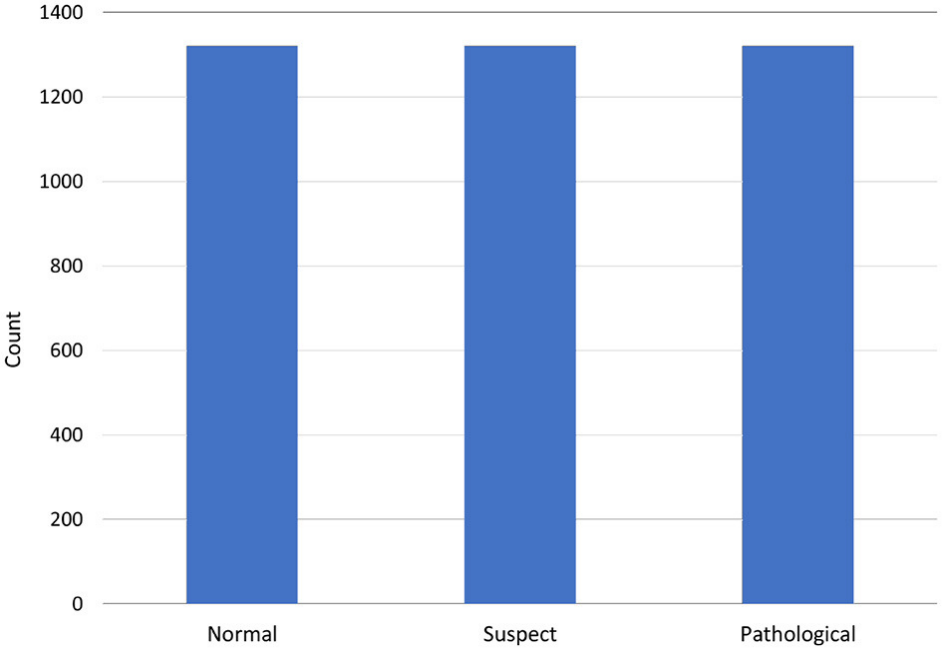
Class-wise dataset distribution after applying SMOTE data upsampling.

Before applying the SMOTE oversample technique, we made a dataset division of 80%–20% which means 20% of the entire dataset is used for testing and 80% for training. From the 20% testing dataset (426 records), healthy fetuses records are 333, suspected 64, and pathological 29. After this training division of 80% (1,700 records), healthy fetus records are 1,322 as compared to 147 pathological and 231 suspected. Then we applied SMOTE data oversampling on this training dataset division and upsampled all classes to 1,322 samples. In this way, our transformed dataset shape becomes (1,322 × 3 = 3,966) samples with 22 features in total. Then on this transformed dataset we trained our models and tested on 426 (20% of testing data) that we split before applying SMOTE oversampling. Figure 3 shows data balancing after applying SMOTE.

### 3.4 Machine learning models for fetal health classification

In this section, we provide a brief description of the classification methods utilized in the study, along with details on the calibration process of the classifier. The following machine learning techniques were employed in the current study, chosen for their widespread use in fetal health classification and other tasks. All models were implemented using scikit-learn. The section includes a concise overview of each model and its corresponding hyperparameters.

#### 3.4.1 Light gradient boosting machine

LightGBM is a swift, efficient, and distributed gradient-boosting structure DT-embedded algorithm. LightGBM is extensively employed in boosting algorithms across multiple ML tasks like classification, ranking, and regression (30). Boosting methods yield a powerful learning model by combining multiple weak ML algorithms. Through successive iterations, the importance of misclassified data points is enhanced while that of correctly classified diminished by such boosting algorithms. A greater intention is ensured by this iterative process to the misclassified classifier in the subsequent training sessions. Eventually, all individual ML models are linearly united, with adjustments made to the combined model weights based on the classifiers' error rates. LightGBM core concept is symbolized in Equation 1.


(1) $$\begin{array}{l} f\left(x\right)=\overset{Q}{\underset{q=1}{\sum }}\alpha_{q}T\left(x,\theta_{q}\right) \end{array}$$

where *f*(*x*) represents the training sample *x* corresponding target value, *Q* denotes base learners' numbers, α_*q*_ signifies the weighted coefficient of the *q*-th base learner, *x* denotes the drilling example, θ_*q*_ represents the learner's classification parameters, *T*(*x*, θ_*q*_) stands for the *q*-th base learner involved in the preparing process.

#### 3.4.2 Random forest classifier

Being an ensemble learning procedure, RF is very proficient at both regression and classification tasks (31). It harnesses the collective strength of numerous decision trees alongside a technique called Bootstrap and Aggregation, or bagging. This approach involves randomly selecting rows and features from the dataset to generate sample datasets for each tree a process known as Bootstrap. Subsequently, the Aggregation step consolidates the predictions of all individual trees to yield the ultimate results. While RF constructs multiple decision trees and averages their predictions, methods like gradient boosting (GB) and XGBoost build models sequentially to rectify the errors of preceding models. RF demonstrates proficiency with unseen data, exhibits reduced susceptibility to overfitting, and maintains computational efficiency. The ultimate forecast is presented as follows.


(2) $$\begin{array}{l} \gamma \left(predicted\right)=\frac{1}{N}\overset{N}{\underset{f=1}{\sum }}Mi \end{array}$$

In the provided equation, *M*_*i*_ represents the prediction of the *i*-th decision tree, and *N* denotes the total number of decision trees in the Random Forest ensemble.

#### 3.4.3 Logistic regression

LR is a performance-enhancing combined multiple RF and AdaBoost-like classifiers type of ensemble learning using an iterative ensemble approach. Consequently, a strong classifier is constructed by it (32). LR identifies the correlation between the categorical independent and dependent variables. Additionally, it computes the posterior probability *p* by fitting the data into the logistic function of an event. The classifier's weights and sample training are fixed in each successive iteration aimed at boosting the basic underlying concept to correctly ascertain the target class of the provided data. LR's classification y^*^ is illustrated as follows:


(3) $$\begin{array}{l} y^{*}=ln\left(\frac{p}{1-p}\right) \end{array}$$

#### 3.4.4 Decision tree classifier

DT represents a non-parametric supervised ML approach utilized to establish classification systems based on multiple covariates or to develop prediction algorithms for a target variable (33). This method involves constructing a model capable of predicting the value of a target variable through the learning of simple decision rules derived from data features. An inverted tree structure is formed after the dataset is organized into branched segments, consisting of internal, root, and leaf nodes. Being a non-parametric algorithm, complex and big datasets are easily handled by it without the need for a complex parametric structure. In the case of the enormous data samples, the data is organized as test and training samples. The training data is then used to build a DT prototype while the test sample determines the appropriate tree size for optimal final model attainment.

#### 3.4.5 Extra trees classifier

Extremely randomized trees or extra trees, are part of the ensemble learning procedures category, similar to RF, where multiple individual DT results are aggregated (34). ETs are superior in performance in comparison to RF algorithms. The baseline difference between ET Regressor and RF is the utilization of bootstrap aggression, which is used by RF while ET doesn't. Instead, it uses the entire training dataset to construct its DTs. ET Regressor, instead of determining the best-split point after all features are taken into consideration, selects features' subset randomly, and eventually a random split point is selected. Overfitting in the model is mitigated by reducing the variance aided by this added randomness. The benefits of ET regressor are proven when datasets are high-dimensional and computational efficiency is a priority.

#### 3.4.6 Extreme gradient boosting

XGBoost employs an objective function comprising a regularization term and a loss function. The discrepancy between expected and actual values of a model is measured using the loss function while overfitting and complexity of the model are managed by the regularization term (35). Notably, XGBoost incorporates parallel processing methods, facilitating quicker computation. In contrast, traditional Gradient Boosting trains each new model sequentially, correcting errors from the preceding model. XGBoost represents a refined version of Gradient Boosting, integrating sophisticated regularization methods and streamlined tree construction processes.

#### 3.4.7 Ada boost classifier

AdaBoost leverages the boosting principle to generate a strong classifier from weaker ones. By integrating subpar classifiers and extracting their predictive value, AdaBoost enhances the overall efficacy of machine learning classifiers, forming a superior ensemble classifier (36). This approach mitigates issues associated with overfitting and contributes to improved results. AdaBoost meticulously evaluates the optimal contributions of each individual classifier, thereby selecting the most effective values for inclusion in the ensemble.

#### 3.4.8 Gradient boosting classifier

Gradient Boosting serves as an ensemble meta-estimator comprising weak prediction models, often decision trees, commonly employed for classification tasks (37). The algorithm predicts classes based on a weighted majority vote of the predictions from the weak learners, with weights determined by their respective accuracy rates. Notably, Gradient Boosting is applicable even to small samples, ranging from as few as 100–1,000 data points.

#### 3.4.9 Support vector machine—linear kernel

SVM is a prominent and preferred supervised algorithm dealing with both the classification and regression tasks, yet it stands out when it comes to classification circumstances (38). In the realm of N-dimensional spaces, SVM algorithms aim to identify a hyperplane that optimally segregates the two classes. In simpler terms, if there are only two input features, this hyperplane reduces to a line if there are more features, it becomes a multidimensional plane. The ideal scenario occurs when the data are perfectly separable, resulting in maximum distance between the nearest class elements and the hyperplanes. SVM endeavors to approximate this scenario as closely as possible. Nonlinear SVM introduces different classes of manifolds as an alternative to hyperplanes, yet the underlying rule remains consistent.

While linear SVM kernels are commonly employed to delineate class labels using lines, not all datasets exhibit linear separability. Kernel functions address this challenge by projecting data points from the original space to a feature space, thereby enhancing separability. However, kernel functions have limitations; they may not be universally applicable across all datasets, and the transformation process itself can be computationally expensive, leading to heightened training and prediction costs.

#### 3.4.10 K nearest neighbors classifier

The KNN algorithm is an extensively used procedure in ML for both the regression and the classification tasks (39). It derived its basis from the fact that similar values or labels are shared by the data points having related features. The entire training dataset is stored as a reference during the training stage. Distance between all training examples and input data points is computed while making predictions, characteristically Euclidean distance-like distance metric is selected. Afterward, the K nearest neighbors are identified by the algorithm based on their distances to the input data point. When it comes to classification tasks, the most common class label among the K neighbors is assigned by the KNN as the input data point's anticipated label. Furthermore, the value of the input data point is predicted by either calculating the weighted averages or the simple averages of K neighbors' target values for the regression tasks.

#### 3.4.11 Naive Bayes

NB function operates on the principles of Bayes' theorem and adopts a conditional probability model. Its fundamental assumption is that every pair of features is independent (40). Employing a supervised learning approach, NB classifies outcomes. Notably, NB necessitates only a small amount of data to construct the model. In determining the probability of mode selection, it is assumed that mode choice adheres to a Gaussian distribution.

#### 3.4.12 CatBoost classifier

CatBoost or Categorical Boosting, Yandex developed an open-source library, specifically tailored to address regression and classification challenges involving numerous independent features (41). A distinguishing feature of CatBoost is its capability to manage both categorical and numerical features seamlessly, eliminating the need for feature encoding techniques and thereby streamlining data preprocessing efforts. Moreover, CatBoost automatically scales all features internally to a suitable range, a feature absent in traditional boosting algorithms. The trained model's overall performance is augmented and faster convergence is facilitated by this.

### 3.5 Evaluation parameters

The proposed model's efficiency and reliability are ensured and demonstrated using various evaluation parameters like accuracy, recall, precision, F1 score, MCC, Kappa, and AUC are among the commonly utilized performance metrics to gauge the performance of similar models. These metrics are comprehensively used to evaluate the performance of the model across various aspects, allowing for a thorough assessment of its reliability and efficacy.

Accuracy is a metric linked to the model's ability to predict the results correctly (42). It is essential to first determine the true negatives (TN), false positives (FP), true positives (TP), and false negatives (FN) when computing accuracy. Using these elements, the following equation is used to calculate the accuracy.


(4) $$\begin{array}{l} Accuracy=\frac{\text{No, of CPr}}{CPr+FPr} \end{array}$$

This formula expresses accuracy as the sum of true outcomes (either positives and/or negatives) divided by the total number of observations (the sum of true positives, false positives, true negatives, and false negatives).

Precision, in the context of classification models, can indeed be computed by determining the proportion of correctly predicted to the total predicted positive observations (43). The equation to calculate precision is as follows.


(5) $$\begin{array}{l} Precision=\frac{TP}{TP+FP} \end{array}$$

Furthermore, in order to demonstrate the number of genuine positive cases our model can predict accurately, we will calculate recall (44). Recall is determined by the ratio of correctly predicted positive observations to all observations in the actual class, as indicated by the following equation.


(6) $$\begin{array}{l} Recall=\frac{TP}{TP+FN} \end{array}$$

Through the computation of precision and recall, we derive the F1 score, another valuable metric used for evaluating model performance (45). The F-score is obtained by taking the weighted average of precision and recall, as described by the following equation:


(7) $$\begin{array}{l} F1Score=2\times \frac{Precision\times Recall}{Precision+Recall} \end{array}$$

The AUC assesses the quality of the model predictions, ranging from zero to one, where a score of 1 indicates the best performance and 0 indicates the worst (46). Furthermore, AUC reflects the degree of separability, elucidating the model's ability to differentiate between classes.

The Kappa is a statistics-based prominent evaluation metric denoted by k, which measures the reliability in relation to other evaluators or parameters (47). The following equation computes the value of *k*.


(8) $$\begin{array}{l} k=\frac{prob\left(O\right)-prob\left(C\right)}{1-prob\left(C\right)} \end{array}$$

In this context, *O* represents the likelihood of observed agreements among the evaluators, while *C* signifies the prospect of agreements anticipated by chance. If *k* = 1, it indicates complete agreement among the evaluators, conversely, if *k* = 0, it suggests no agreement among the evaluators.

MCC is an unaffected and substitute measure for uneven datasets and employs a likelihood matrix method to calculate the Pearson product-moment correlation coefficient between predicted and actual values (48). Expressed in terms of the entries of the contingency matrix M, MCC is formulated as follows:


(9) $$\begin{array}{l} MCC=\frac{TP.TN-FP.FN}{\left(TP+FP\right).\left(TP+FN\right).\left(TN+FP\right).\left(TN+FN\right)} \end{array}$$

The MCC is unique among binary classification metrics in that it yields a high score only when the binary predictor successfully predicts the majority of positive and negative data instances.

## 4 Experiments and analysis

The experiments were performed using Python 3.8, and TensorFlow and Scikit-learn libraries. The experimental setup operated with 8GB of available RAM, on Windows 10 (64-bit) operating system. The CPU utilized was 7th Gen, Intel Core i7 having a clock speed of 2.8 GHz, and an 8 GB, GTX 1060 GPU from Nvidia. Technical specifications of the computational resources deployed in research are crucial for the in-depth understanding of the model. Table 2 summarizes the experimental setup.

**Table 2:** Framework specifications.

**Element**	**Details**
Programming environment	Python 3.8
Software libraries	Scikit-learn, TensorFlow
OS	Windows 10 (64-bit)
RAM	8GB
Processor	7th Gen, Intel Core i7, 2.8 GHz processor
Graphics card	8 GB- GTX 1060 Powered GPU from Nvidia

### 4.1 Results of machine learning models on CTG dataset for fetal health

Extensive experiments were conducted for fetal health detection, wherein various machine learning models were applied under different scenarios. These models were optimized concerning various hyperparameters for better performance. Furthermore, the results were examined by employing individual machine-learning models across all feature sets in the experiments. The results of all learning models are shared in Table 3.

**Table 3:** Classification report of all learning models.

**Model**	**Accuracy**	**AUC**	**Recall**	**Prec**.	**F1**	**Kappa**	**MCC**	**TT (s)**
LightGBM	0.9989	0.9988	0.9832	0.9834	0.9832	0.9748	0.9749	2.8470
CB	0.9766	0.9984	0.9766	0.9770	0.9766	0.9649	0.9651	8.4650
ETC	0.9762	0.9987	0.9762	0.9769	0.9762	0.9643	0.9647	0.9380
XGBoost	0.9748	0.9984	0.9748	0.9752	0.9747	0.9622	0.9624	2.0540
RF	0.9708	0.9982	0.9708	0.9713	0.9708	0.9562	0.9565	1.2220
GBC	0.9647	0.9969	0.9647	0.9656	0.9648	0.9470	0.9474	4.1070
DT	0.9510	0.9633	0.9510	0.9516	0.9510	0.9265	0.9268	0.2000
KNN	0.9416	0.9893	0.9416	0.9455	0.9416	0.9125	0.9143	0.4690
Ada	0.9121	0.9673	0.9121	0.9140	0.9123	0.8682	0.8689	0.7400
LR	0.8897	0.9735	0.8897	0.8935	0.8904	0.8346	0.8358	0.3100
SVM	0.8768	0.0000	0.8768	0.8815	0.8775	0.8152	0.8167	0.1710
NB	0.7677	0.8925	0.7677	0.8223	0.7708	0.6515	0.6757	0.1700

The provided information outlines the performance metrics of various machine learning models across multiple evaluation criteria. Each model undergoes assessment based on AUC, MCC, recall, precision, F1 score, accuracy, kappa score, and training time in seconds. These metrics collectively offer insights into how effectively each model performs in a classification task.

Beginning with the top-performing models, LightGBM achieves notable results with an accuracy of 99.89% and an AUC of 99.88%, showcasing its proficiency in correctly classifying instances and distinguishing between classes. Additionally, its recall, precision, and F1 score of 97.91% indicate its ability to identify positive instances accurately while minimizing false positives. Moreover, the kappa score and MCC of ~96.80% further validate the model's reliability. Similarly, the CatBoost classifier and ETC demonstrate strong performance with high accuracy of 97.66% and 97.62%, respectively, and AUC values of 99.84% and 99.87%, respectively. These models maintain recall, precision, and F1 scores above 97%, reflecting their effectiveness across various evaluation metrics. Despite slight variations in training times of 8.465 s for CatBoost and 0.938 s for ETC, both models exhibit robust performance in classification tasks.

The XGBoost and RF classifier also deliver commendable results with accuracy and AUC values exceeding 97% and 99%, respectively. Leveraging ensemble methods, these models harness multiple decision trees to achieve strong performance, as evidenced by their high recall, precision, and F1 scores. Furthermore, they boast reasonable training times, making them efficient choices for practical applications.

Descending through the list, the GBC, DT classifier, and KNN classifier demonstrate good performance with accuracy scores ranging from 96.47% to 95.10%. Although their AUC values are slightly lower compared to the top performers, they still exhibit respectable recall, precision, and F1 scores, underscoring their efficacy in classification tasks.

Further down the spectrum, the LR, SVM with a linear kernel, AdaBoost classifier, and NB show diminishing performance in terms of accuracy, AUC, and other evaluation metrics. While these models offer reasonable results, they may not be as suitable for tasks requiring high precision or discrimination between classes. Notably, the SVM with a Linear Kernel exhibits an AUC of 0.00%, indicating potential issues with its discriminative ability in the given dataset, highlighting the importance of meticulous model selection and parameter tuning to ensure optimal performance.

**Figure 4:**
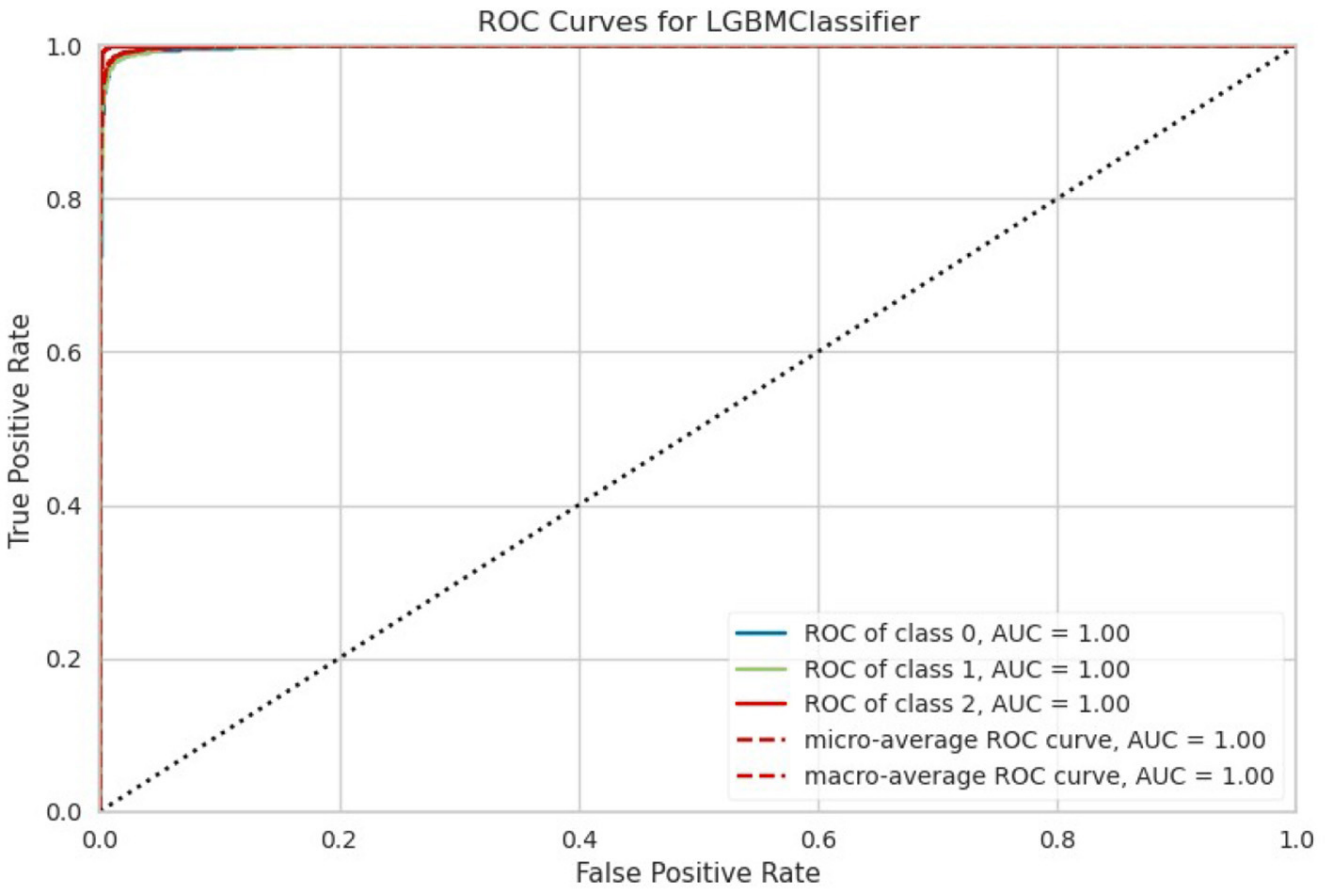
ROC-AUC curve.

In summary, the data provides a comprehensive overview of the performance of different machine learning models in a classification task. Ensemble methods like LightGBM, CatBoost, ETC, XGBoost, and RF classifier excel in terms of accuracy, AUC, and overall robustness, as shown in Figure 4. However, the selection of a model should consider not only performance metrics but also dynamics like computational efficiency, interpretability, and explicit task specifications to ensure the most suitable model is chosen for deployment.

**Figure 5:**
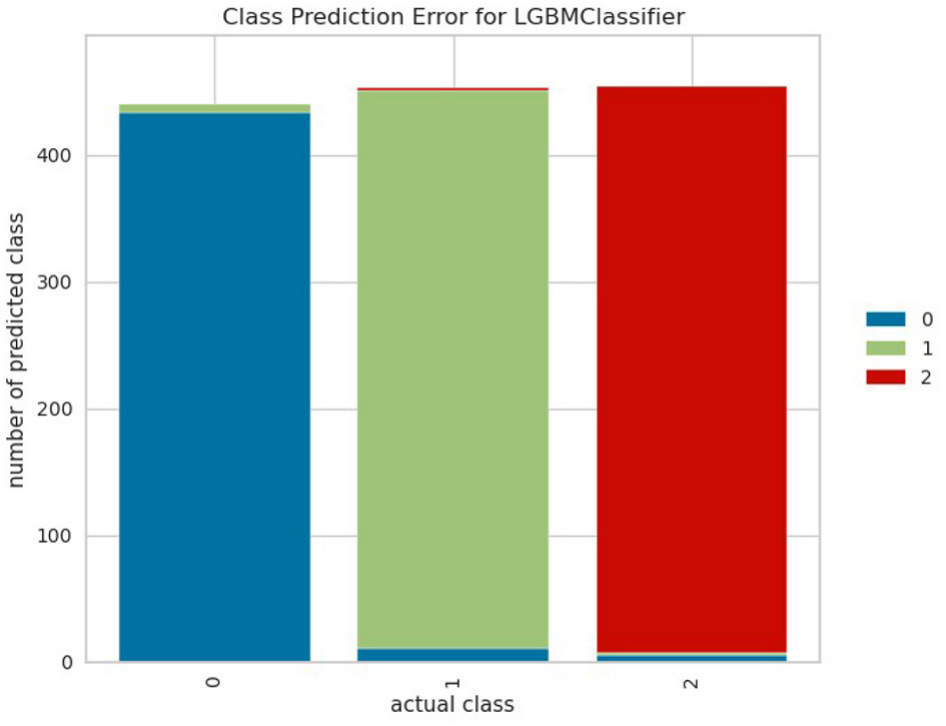
Analysis of error rate.

**Figure 6:**
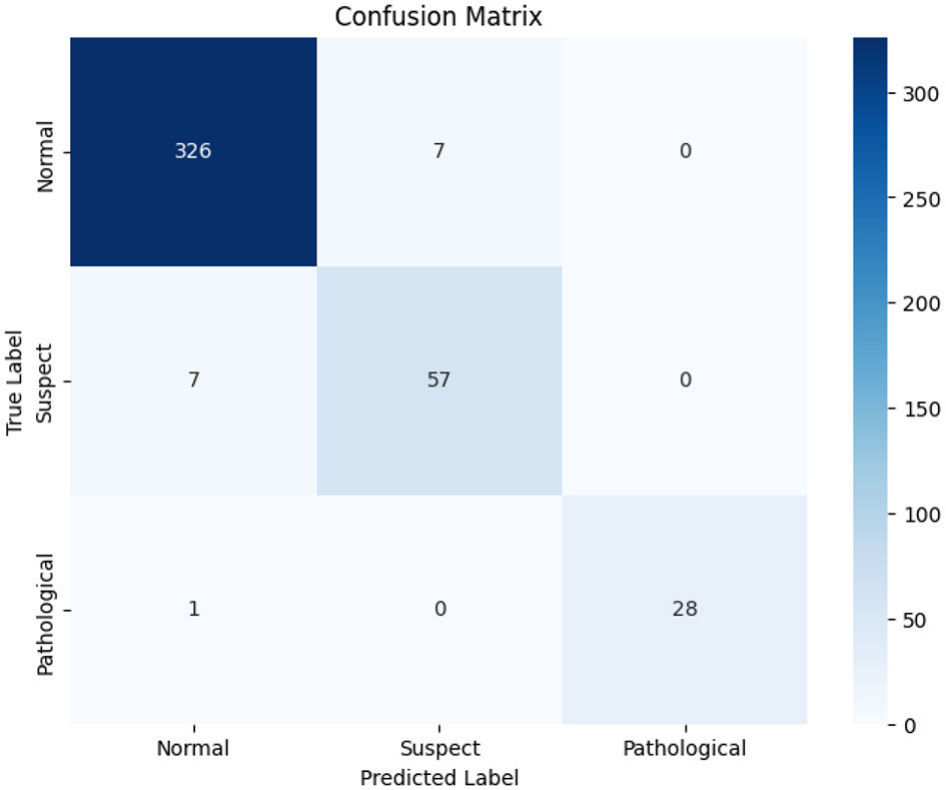
Confusion matrix for the LightGBM model.

Figure 5 shows the class-wide prediction error rate from the proposed LightGBM model indicating that the model shows a similar performance for all the classes used for experiments. In addition, the number of wrong predictions is very few. The same can be confirmed by the confusion matrix shown in Figure 6 which shows the number of wrong predictions. The model makes a total of 15 wrong predictions indicating better performance of the proposed LightGBM compared to other models used in this study.

### 4.2 Interpretation and explainable analysis of results using SHAP

**Figure 7:**
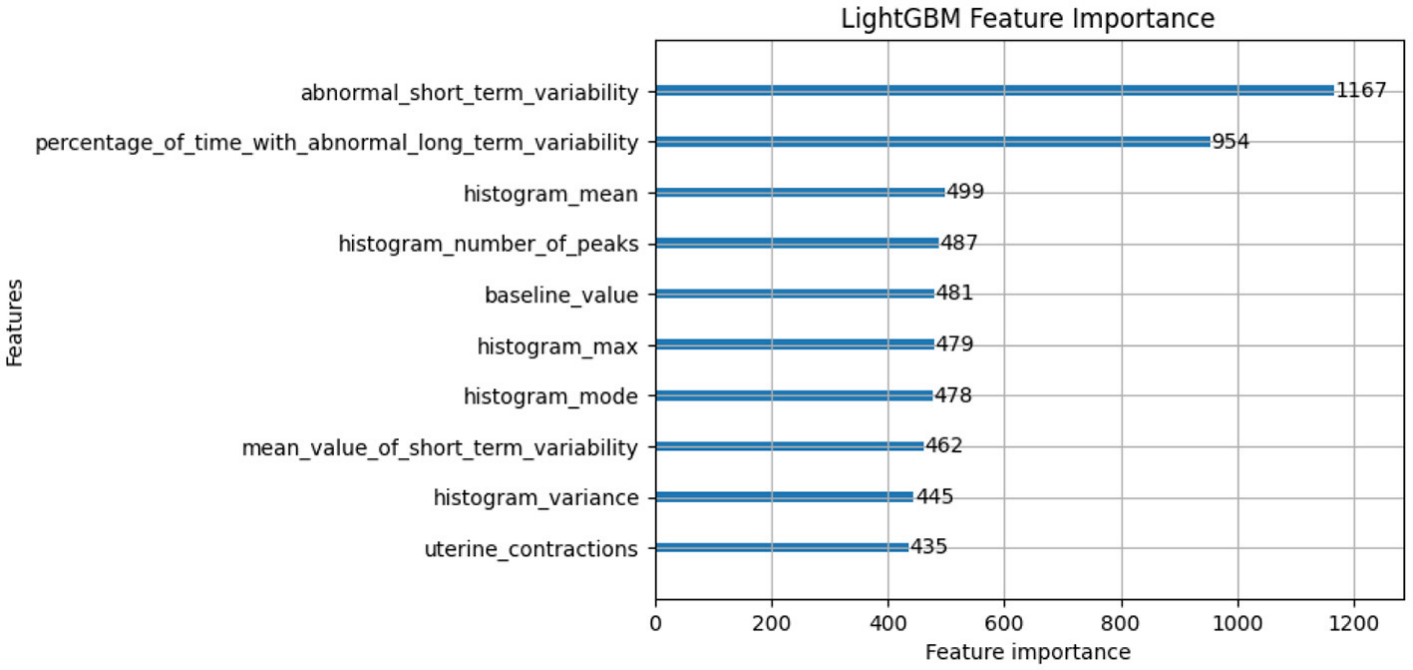
Feature importance of LightGBM model.

In this subsection, we will discuss the LGBM model feature importance results and Exaplainable AI SHAP summary and dependency results of each class and analyze what each feature describes (49). The feature importance of the LGBM model is shown in Figure 7. The feature importance plot gives us an indication of which features have the most significant impact on the model's predictions. The interpretation of feature importance shown in Figure 7 is “abnormal_short_term_variability” feature has the highest importance score, meaning it has the most significant impact on predicting fetal health. Short-term variability in fetal heart rate is known to be a strong indicator of fetal distress or abnormal conditions. The “percentage_of_time_with_abnormal_long_term_variability” is another key feature, which likely tracks the percentage of time during which the fetal heart rate exhibits abnormal long-term patterns. Long-term variability is crucial for assessing the fetus's health. Other features like “histogram_mean,” “histogram_number_of_peaks,” and “baseline_value” are related to heart rate statistics and also play a significant role in classification. They likely capture the overall distribution and trends in fetal heart rates, which are critical for monitoring fetal wellbeing. Other notable features include “histogram_max,” “mean_value_of_short_term_variability,” and “uterine_contractions.” All of these are highly relevant to assessing the fetus's response to external stimuli or stress during pregnancy.

**Figure 8:**
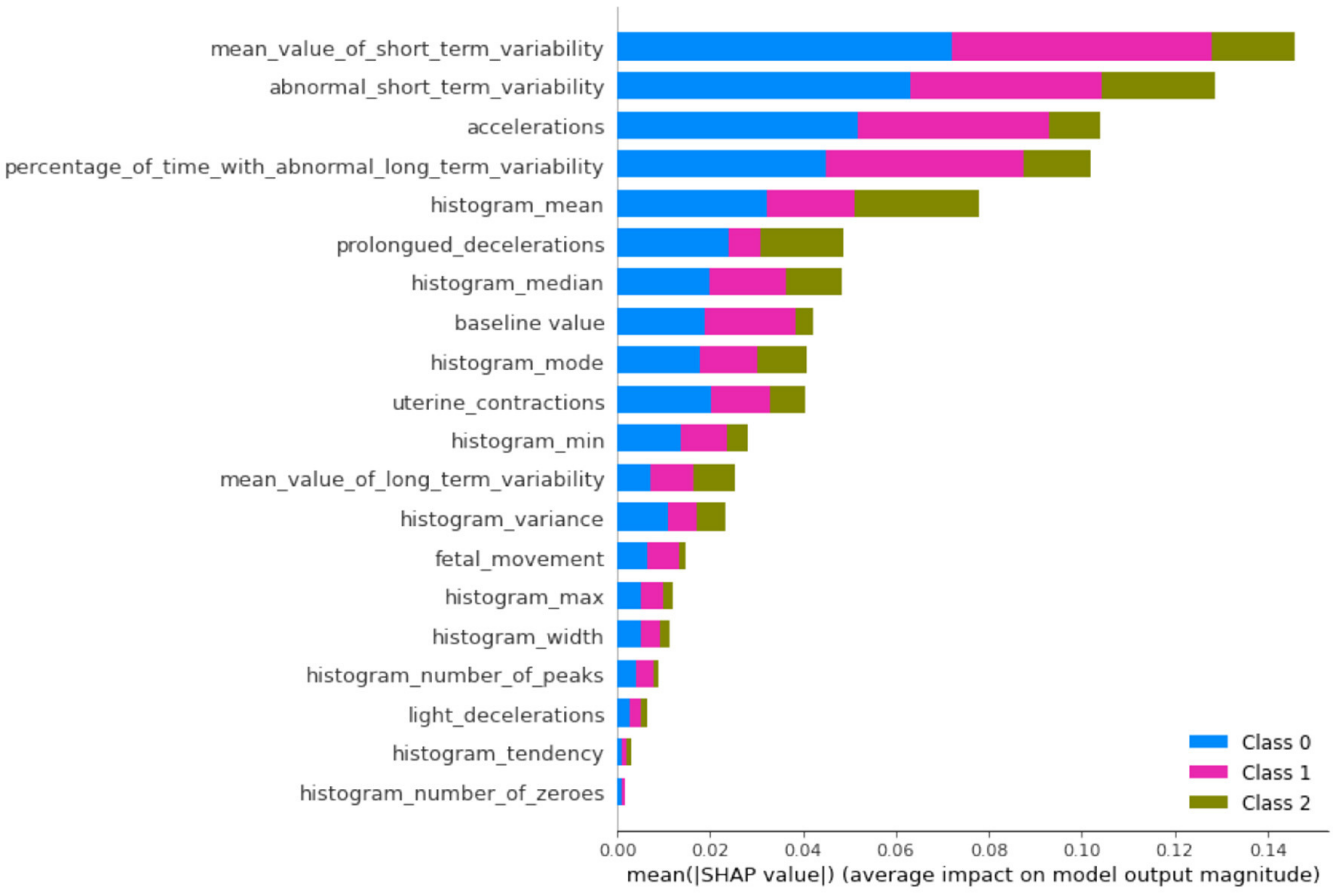
Overall summary of SHAP XAI.

The SHAP plots allow us to interpret how each feature impacts individual predictions. Positive SHAP values push the model toward predicting a certain class (e.g., a good fetal condition), while negative values push toward the opposite class. The color gradient (red to blue) represents the feature value, with red indicating high feature values and blue indicating low feature values. Again the importance feature “abnormal_short_term_variability” high values (in red) tend to increase the SHAP value, pushing the model toward predicting abnormal fetal health. This aligns with clinical knowledge, as greater variability is often indicative of fetal distress. The second-ranked feature “percentage_of_time_with_abnormal_long_term_variability” high values of long-term variability push the model toward predicting abnormal conditions. The features “prolongued_decelerations” and “accelerations” in fetal heart rate are critical markers for predicting fetal health. In this case, abnormal values increase the likelihood of predicting fetal issues. It can also be observed from Figure 8, where overall SHAP summary is explained that the lower impact features are “histogram_mean,” “baseline_value,” and “histogram_median” have more nuanced impacts. Their effects are smaller but still crucial for the model. For example, a low “baseline_value” (blue dots) typically pushes the model toward predicting normal fetal health, while higher baseline values (red dots) lean toward predicting abnormality.

**Figure 9:**
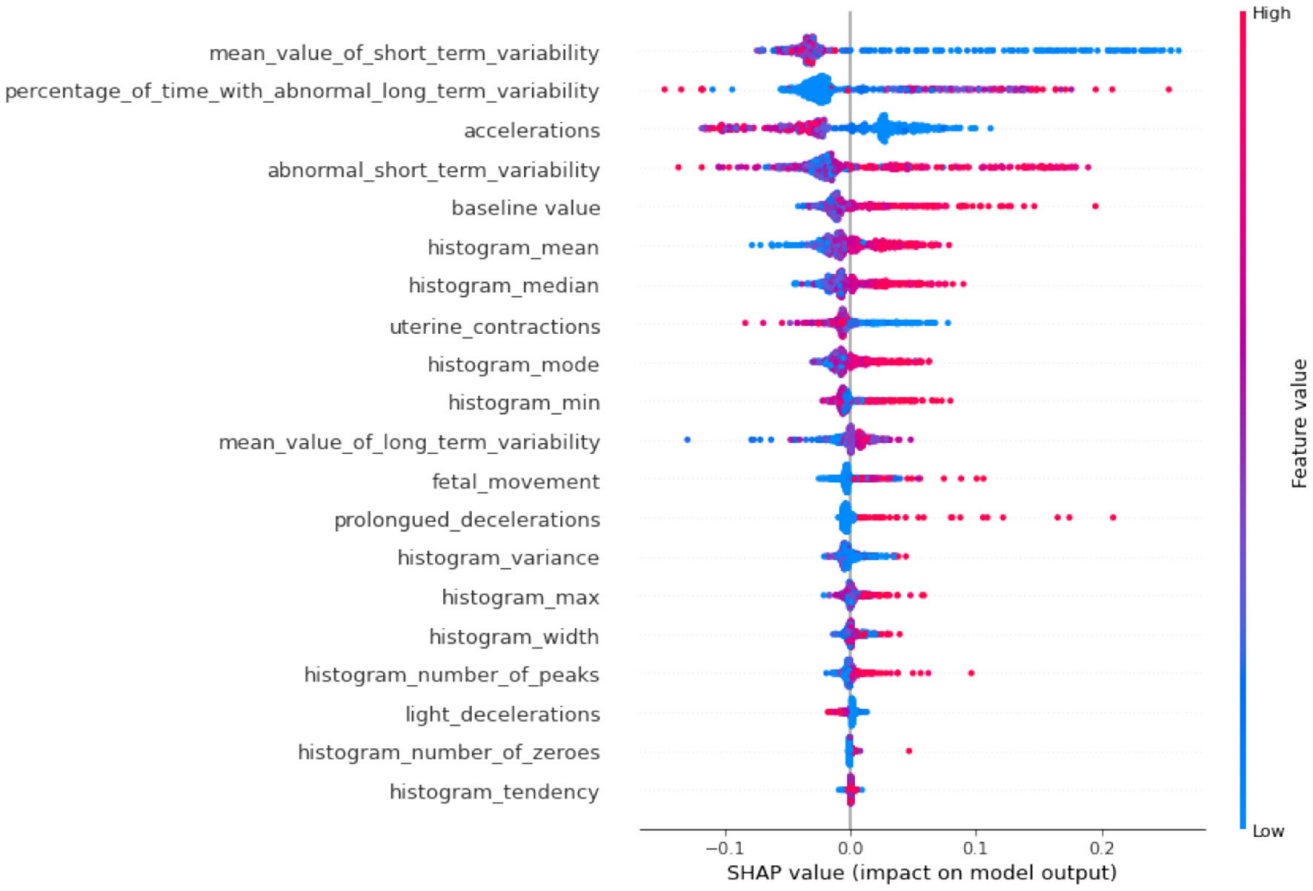
SHAP summary of pathological class.

**Figure 10:**
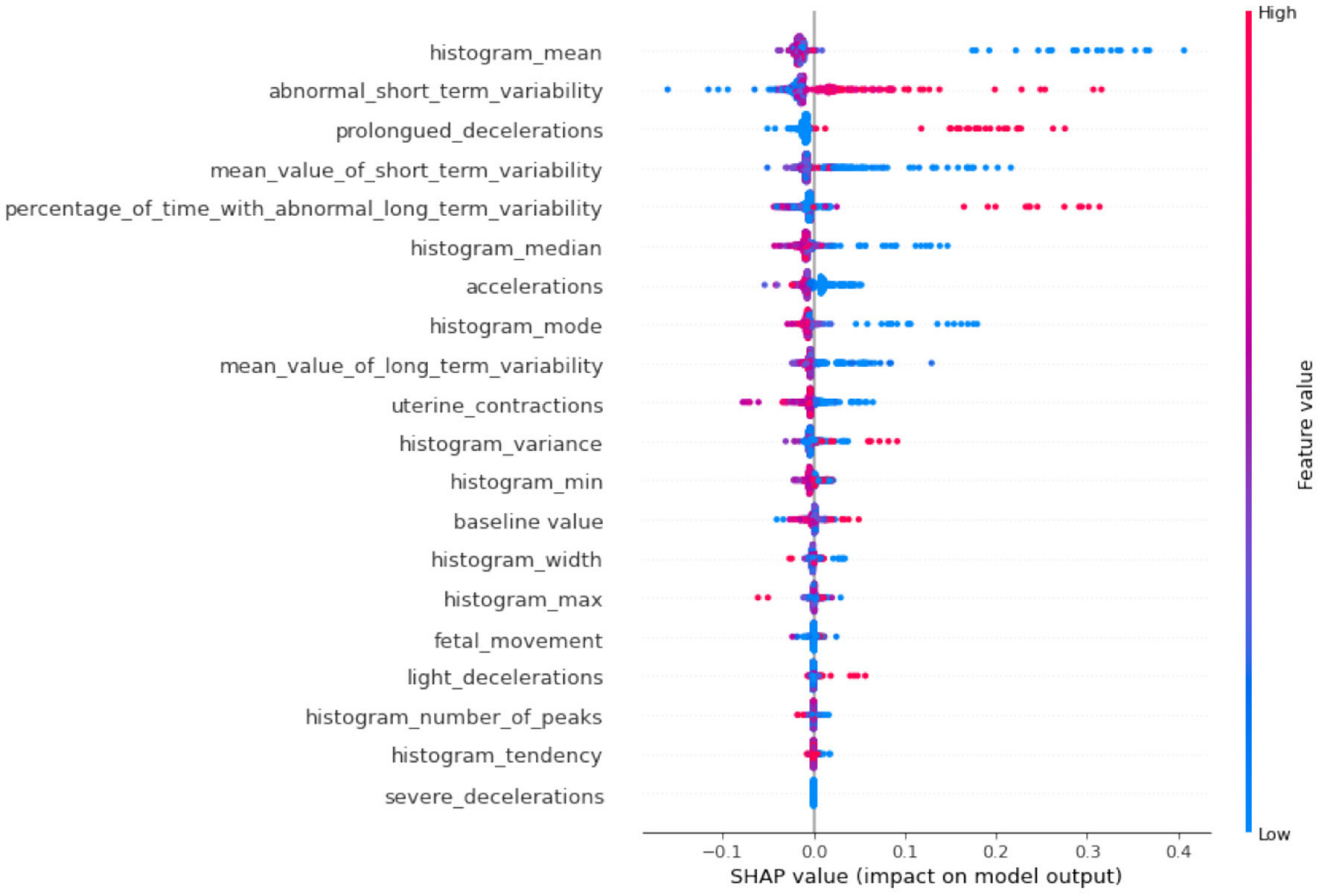
SHAP summary of suspect class.

**Figure 11:**
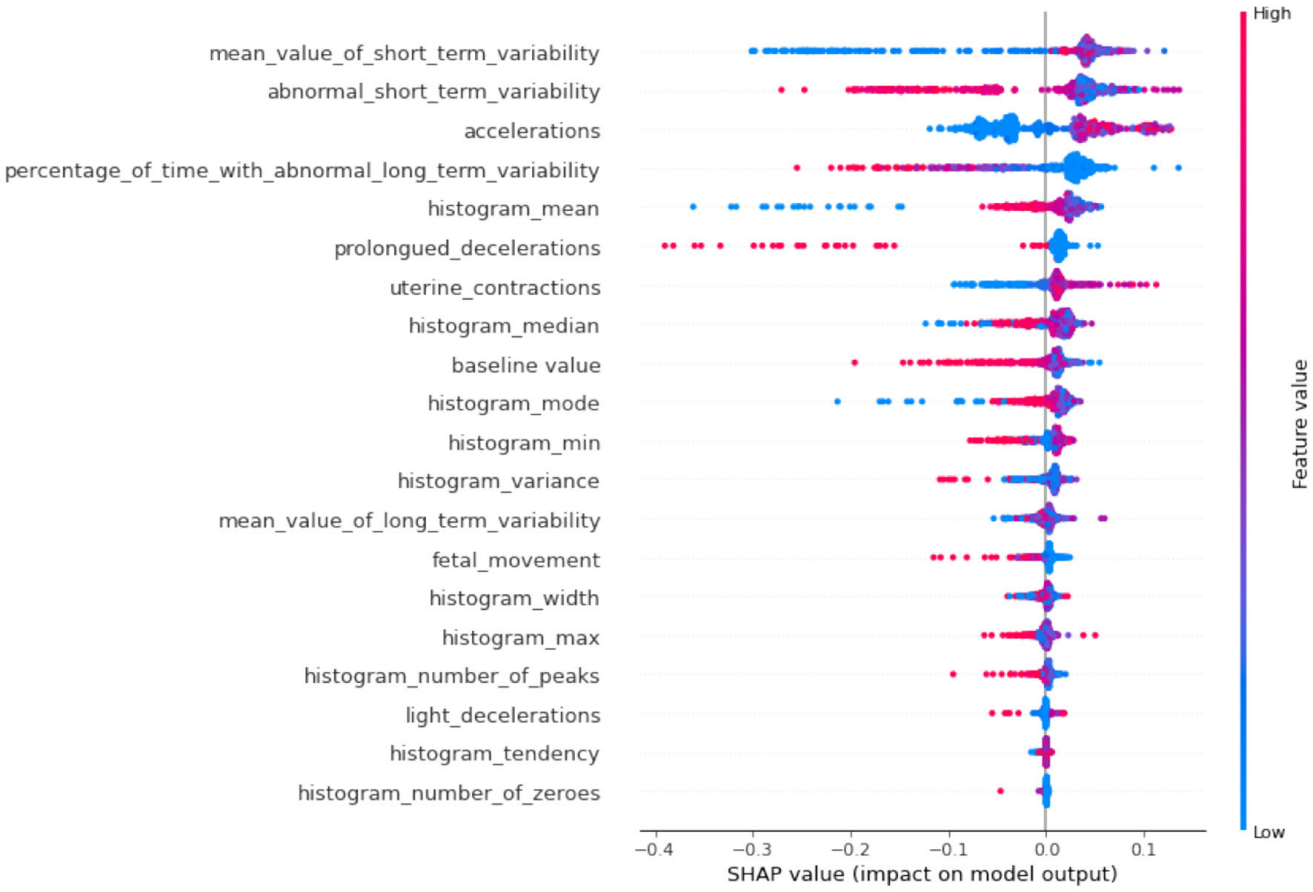
SHAP summary of normal class.

If we compare SHAP pathological (Figure 9) vs. SHAP suspect (Figure 10), comparing the two SHAP plots, we see similar features driving predictions, with slight differences in order. This likely reflects different aspects of the model's behavior under various conditions (e.g., distinguishing between normal and abnormal health states). The interaction between features (such as short-term variability with other heart rate metrics) also becomes evident in these plots. The sHAP explanation of the normal target class is shown in Figure 11.

### 4.3 K-fold cross-validation results

The proposed model's performance is validated using ten-fold validation. Its purpose is to validate and ascertain the robustness of the generated results by the recommended model. The cross-validation process ensures the performance consistency of the model across all data subsets. In this study, 10-fold cross-validated results are summarized in Table 4. The suggested LGBM model exhibits a 97.91% average accuracy score in cross-validation results. Additionally, the average recall, precision, and F1 scores are found to be 97.91%, 97.93%, and 97.91%, respectively. In 10-fold cross-validation, LGBM also attains an AUC value of 99.88%, Kappa score of 96.86, and MCC of 96.88. The standard deviation of accuracy for LGBM across the 10 folds is calculated to be 0.0088.

**Table 4:** 10-fold cross-validation results concerning various metrics.

**Fold**	**Accuracy**	**AUC**	**Recall**	**Precision**	**F1**	**Kappa**	**MCC**
0	0.9928	0.9995	0.9928	0.993	0.9928	0.9892	0.9893
1	0.9712	0.9982	0.9712	0.972	0.9714	0.9568	0.9571
2	0.982	0.9985	0.982	0.982	0.982	0.973	0.973
3	0.9784	0.9988	0.9784	0.9787	0.9785	0.9676	0.9678
4	0.9892	0.9999	0.9892	0.9892	0.9892	0.9838	0.9838
5	0.9784	0.9973	0.9784	0.9786	0.9784	0.9676	0.9677
6	0.9856	0.9997	0.9856	0.9858	0.9855	0.9783	0.9785
7	0.9603	0.9983	0.9603	0.9608	0.96	0.9404	0.9409
8	0.9747	0.9992	0.9747	0.9747	0.9747	0.9621	0.9621
9	0.9783	0.9985	0.9783	0.9786	0.9784	0.9675	0.9676
Mean	0.9791	0.9988	0.9791	0.9793	0.9791	0.9686	0.9688
Std	0.0088	0.0008	0.0088	0.0087	0.0089	0.0132	0.0131

### 4.4 Comparison of performance with existing studies

The recommended prototype's performance is evaluated against previous advanced models by conducting a performance comparison in-between them. To demonstrate the performance of the projected model in comparison to previous advanced models, a comparison with existing models is conducted. This research selects the nine most relevant previous studies for this purpose. For instance, Yin and Bingi (17) utilized the SVM ML model for fetal health classification, and a 99.59% impressive accuracy score was obtained. In another study (23), the GBC was employed, achieving the highest accuracy score of 95%. T2-FNN was utilized by Abiyev et al. (18), resulting in an accuracy score of 96.66%. Additionally, Sudharson et al. (26) utilized DT resulting in 90.8% accuracy. Likewise, Afridi et al. (24) and Salini et al. (25) utilized the RF model and NB models, with 93% and 83.06% accuracy, respectively. Table 5 illustrates the performance comparison between the proposed model and existing studies, revealing the superiority of the proposed model.

**Table 5:** Performance comparison with existing studies.

**References**	**Classifier**	**Accuracy**
Yin and Bingi (17)	SVM	99.59%
Abiyev et al. (18)	T2-FNN (63 rules)	96.66%
Kuzu and Santur (19)	XGB	99.10%
Muhammad Hussain et al. (20)	AlexNet-SVM,	99.72%
Piri and Mohapatra (21)	XGBoost and RF	94.32%
Al Duhayyim et al. (22)	XGB, CatBoost, VC	99.05%
Islam et al. (23)	GBC	95.19%
Afridi et al. (24)	NB	83.06%
Salini et al. (25)	RF	93.15%
Sudharson et al. (26)	DT	90.80%
**Proposed**	**LGBM**	**99.89%**

Bold values indicating the results of proposed model which is better than all SOTA models.

When we compare the proposed LightGBM model with the traditional approaches and potential models used for fetal health monitoring, several benefits of the proposed model can be observed. In terms of real-time performance and computation, LightGBM has proved to be faster than other models such as SVM, AlexNet, RF, FNN, and XGBoost utilized in previous research works. This also gives it an advantage in dealing with diverse numerical feature datasets which is a crucial area of consideration given the ever-increasing nature of medical data. LightGBM's built-in regularization mechanisms help prevent overfitting, ensuring strong generalization to unseen data. The model also provides a detailed feature importance analysis itself and through techniques such as SHAP thereby helping clinicians understand the model's decisions. In this research work, we utilized and shared both information (obtained through the feature importance of LGBM and by SHAP dependency graphs). As opposed to most of the other frameworks, LightGBM can identify rather intricate interactions between specific fetal health characteristics and other factors and has good generality. Furthermore, it reduces human error in prognosis analysis by detecting subtle variations that may be missed during manual assessments, leading to more accurate diagnoses. This combination of efficiency, accuracy, and interpretability makes the proposed model superior to existing clinical standards and machine learning models.

### 4.5 Ethical implications, deployment, biases, and data privacy

AI models like LightGBM can introduce biases if the training data is not representative of diverse populations, potentially leading to misdiagnoses or unequal healthcare outcomes. Ensuring diversity in datasets, conducting bias audits, and employing fairness metrics are essential steps to mitigate these risks. Additionally, privacy concerns must be addressed by anonymizing patient data, adhering to regulations like HIPAA or GDPR, and implementing secure data handling practices.

Ethical deployment of AI models requires transparency, explainability, and human oversight. Clinicians should understand how the model makes decisions through tools like SHAP plots, and AI should support (like we have added SHAP plots and feature importance) rather than replace human judgment. Regular monitoring and validation of the model's performance in real-world settings ensure safety and accuracy over time. By incorporating these measures, any research can demonstrate a responsible and ethical approach to AI in fetal and maternal health monitoring.

### 4.6 Implications of proposed approach concerning traditional clinical methods

Traditional clinical methods emphasize diagnosis, prognosis, and treatment (50). Diagnosis has been regarded as the primary element in clinical methods providing classification of subjects into sick and healthy subjects. It is the basis for treatment and prognosis where prognosis determines the future happenings of individuals being classified (51). While all components are important, this study focuses on the diagnosis part of traditional clinical methods due to its importance.

It is important to emphasize that the proposed LightGBM model has been developed with input from a medical professional, a consultant gynecologist who guided the research design. According to her insights, traditional clinical standards such as differential diagnosis methods and prognosis analysis are commonly used by obstetricians and gynecologists. However, these methods are often labor-intensive and prone to human error, particularly when it comes to analyzing complex data over extended periods. The proposed AI-driven model aims to reduce such errors by providing more precise and automated decision support, which can help in the earlier detection of potential complications.

Another doctor highlighted that cardiotocography and ultrasound analysis, despite being widely adopted in fetal monitoring, can sometimes miss critical diagnostic points. Both the patient and doctor might overlook subtle but important signs, leading to potential complications. The integration of LightGBM and data mining techniques offers an opportunity to enhance these traditional methods by identifying patterns that might go unnoticed during manual assessments. This model can therefore supplement traditional approaches, making fetal health monitoring more accurate and less prone to human oversight.

## 5 Conclusions and future work

Fetal health monitoring has substantial importance in saving the lives of pregnant women and fetuses. This research underscores the paramount importance of fetal health in prenatal care and obstetrics, emphasizing its direct impact on both the fetus and the mother. Through continuous monitoring throughout pregnancy, potential risks or complications can be identified and addressed promptly, leading to improved outcomes for both parties. Leveraging cardiotocography data, which encompasses crucial fetal physiological parameters, this study employs a robust methodology for fetal health classification. The utilization of the SMOTE data upsampling technique addresses the class imbalance issue inherent in medical diagnostics, enhancing the model's performance. By employing the LightGBM model, remarkable results are achieved, with high accuracy, AUC, recall, precision, F1 score, Kappa, and MCC values obtained on the test dataset. Comparative analysis against eleven other machine learning models highlights the superiority of the proposed approach in fetal health classification. Furthermore, the significance of the proposed model is investigated through rigorous evaluation using a 10-fold cross-validation and previous research works comparison. Overall, this research contributes to advancing prenatal care by providing an effective and reliable framework for fetal health assessment and classification. The future work direction of this research is to incorporate additional data sources such as ultrasound images, maternal health records, and genetic information as it could provide a more comprehensive understanding of fetal health and improve classification accuracy. In addition, enhancing the interpretability and explainability of the classification model could improve its acceptance and adoption by healthcare providers, facilitating better-informed clinical decisions.

## Data Availability

The original contributions presented in the study are included in the article/supplementary material, further inquiries can be directed to the corresponding authors.

## Footnotes

^1^ https://onlinelibrary.wiley.com/doi/10.1002/1520-6661(200009/10)9:5%3C311::AID-MFM12%3E3.0.CO;2-9

## References

[1] UNICEF. UNCEF, World Health Organization, the World Bank Group, and the United Nations 2019 Levels and trends in child mortality Report 2019. (2019). Available at: https://data.unicef.org/resources/levels-andtrends-in-child-mortality/ (accessed April 12, 2022).

[2] Goldenberg RL Saleem S Pasha O Harrison MS McClure EM. Reducing stillbirths in low-income countries. Acta Obstet Et Gynecol Scand. (2016) 95:135–43. 10.1111/aogs.12817 26577070

[3] Mahdizadeh J Bouraghi H Panahi SSGS Mohammadpour A Shargh AK Mojarad MR . A theory map of the causes of perinatal death in a developing country. Crescent J Med Biol. (2019) 6:237–41.

[4] Ayres-de Campos D Spong CY Chandraharan E. FIGO consensus guidelines on intrapartum fetal monitoring: cardiotocography. Int J Gynecol Obstet. (2015) 131:13–24. 10.1016/j.ijgo.2015.06.020 26433401

[5] Jepsen I Blix E Cooke H Adrian SW Maude R. The overuse of intrapartum cardiotocography (CTG) for low-risk women: an actor-network theory analysis of data from focus groups. Women Birth. (2022) 35:593–601. 10.1016/j.wombi.2022.01.003 35078743

[6] Nazir L Lakhta G Anees K Khan FR Safdar S Nazir GR . Admission cardiotocography as a predictor of low Apgar score: an observational, cross-sectional study. Cureus. (2021) 13:e14530. 10.7759/cureus.14530 34012738 PMC8127024

[7] Nadeem G Rehman A Bashir H. Risk factors associated with birth asphyxia in term newborns at a tertiary care hospital of Multan, Pakistan. Cureus. (2021) 13:e18759. 10.7759/cureus.18759 34796056 PMC8590025

[8] World Health Organization. Perinatal asphyxia. (2022). Available at: https://www.who.int/teams/maternal-newborn-child-adolescent-health-and-ageing/newborn-health/perinatal-asphyxia (accessed January 22, 2024).

[9] World Health Organization. Health Statistics 2022: Monitoring Health for the SDGs, Sustainable Development Goals. (2022). Available at: https://www.who.int/publications/i/item/9789240051157 (accessed January 22, 2024).

[10] Eleftheriades M Pervanidou P Chrousos G. Fetal stress. In:Fink G, editor. Encyclopedia of Stress, 2nd Edn. New York, NY: Academic Press (2007), p. 46–51. 10.1016/B978-012373947-6/00492-X

[11] K H Yu C Bower S. Chapter 10–Fetal growth. In:AM Coady S Bower, editors. Twining's Textbook of Fetal Abnormalities, 3rd Edn. New York, NY: Churchill Livingstone (2015), p. 211–22. 10.1016/B978-0-7020-4591-2.00010-3

[12] Manikandan M Vijayakumar P. Improving the performance of classifiers by ensemble techniques for the premature finding of unusual birth outcomes from cardiotocography. IETE J Res. (2021) 69:1734–44. 10.1080/03772063.2021.1910579

[13] Davidson L Boland MR. Enabling pregnant women and their physicians to make informed medication decisions using artificial intelligence. J Pharmacokinet Pharmacodyn. (2020) 47:305–18. 10.1007/s10928-020-09685-1 32279157 PMC7473961

[14] Sadiq MT Yu X Yuan Z Rehman AU Ullah I Li G . Motor imagery EEG signals decoding by multivariate empirical wavelet transform-based framework for robust brain-computer interfaces. IEEE Access. (2019) 7:171431–51. 10.1109/ACCESS.2019.2956018

[15] Ahmad I Ullah I Khan WU Rehman AU Adrees MS Saleem MQ . Efficient algorithms for E-healthcare to solve multiobject fuse detection problem. J Health Eng. (2021) 2021:9500304. 10.1155/2021/9500304

[16] Moreira MWL Rodrigues JJPC Carvalho FHC Chilamkurti N Al-Muhtadi J Denisov V. Biomedical data analytics in mobile-health environments for high-risk pregnancy outcome prediction. J Ambient Intell Humaniz Comput. (2019) 10:4121–34. 10.1007/s12652-019-01230-4

[17] Yin Y Bingi Y. Using machine learning to classify human fetal health and analyze feature importance. BioMedInformatics. (2023) 3:280–98. 10.3390/biomedinformatics3020019

[18] Abiyev R Idoko JB Altiparmak H Tüzünkan M. Fetal health state detection using interval type-2 fuzzy neural networks. Diagnostics. (2023) 13:1690. 10.3390/diagnostics13101690 37238176 PMC10217653

[19] Kuzu A Santur Y. Early diagnosis and classification of fetal health status from a fetal cardiotocography dataset using ensemble learning. Diagnostics. (2023) 13:2471. 10.3390/diagnostics13152471 37568833 PMC10417593

[20] Muhammad Hussain N Rehman AU Othman MTB Zafar J Zafar H Hamam H. Accessing artificial intelligence for fetus health status using hybrid deep learning algorithm (AlexNet-SVM) on cardiotocographic data. Sensors. (2022) 22:5103. 10.3390/s22145103 35890783 PMC9319518

[21] Piri J Mohapatra P. Exploring fetal health status using an association based classification approach. In: 2019 International Conference on Information Technology (ICIT). Bhubaneswar: IEEE (2019), p. 166–71. 10.1109/ICIT48102.2019.00036

[22] Al Duhayyim M Abbas S Al Hejaili A Kryvinska N Almadhor A Mughal H. Ensemble learning for fetal health classification. Comput Syst Sci Eng. (2023) 47:823–42. 10.32604/csse.2023.037488

[23] Islam MM Rokunojjaman M Amin A Akhtar MN Sarker IH. Diagnosis and classification of fetal health based on CTG data using machine learning techniques. In: International Conference on Machine Intelligence and Emerging Technologies. Cham: Springer Nature Switzerland (2022), p. 3–16. 10.1007/978-3-031-34622-4_1

[24] Afridi R Iqbal Z Khan M Ahmad A Naseem R. Fetal heart rate classification and comparative analysis using cardiotocography data and KNOWN classifiers. Int. J. Grid Distrib. Comput. (2019) 12:31–42. 10.33832/ijgdc.2019.12.1.03

[25] Salini Y Mohanty SN Ramesh JVN Yang M Chalapathi MMV. Cardiotocography data analysis for fetal health classification using machine learning models. IEEE Access. (2024) 12:26005–22. 10.1109/ACCESS.2024.3364755

[26] Sudharson D Vignesh K Dubey AK Mukilan K Kizore MN Ram SA. Impact of classification algorithms on cardiotocography dataset for fetal state prediction. Asian J Comput Sci Eng. (2022) 7:71–6.28679415

[27] LARXEL. Fetal Health Classification. (2020). Available at: https://www.kaggle.com/datasets/andrewmvd/fetal-health-classification (accessed January 5, 2024).

[28] Han H Wang WY Mao BH. Borderline-SMOTE: a new over-sampling method in imbalanced data sets learning. Adv Intell Comput. (2005) 3644:878–87. 10.1007/11538059_91

[29] Chawla NV Bowyer KW Hall LO Kegelmeyer WP SMOTE. Synthetic minority over-sampling technique. J Artif Intell Res. (2002) 16:321–57. 10.1613/jair.953

[30] Ke G Meng Q Finley T Wang T Chen W Ma W . LightGBM: a highly efficient gradient boosting decision tree. Adv Neural Inf Process Syst. (2017) 30:3149–57. 10.5555/3294996.3295074

[31] Breiman L. Random forests. Mach Learn. (2001) 45:5–32. 10.1023/A:1010933404324

[32] Hosmer Jr DW Lemeshow S Sturdivant RX. Applied Logistic Regression, Vol. 398. Hoboken, NJ: John Wiley & Sons (2013). 10.1002/9781118548387

[33] Breiman L Friedman JH Olshen RA Stone CJ. Classification and Regression Trees. Boca Raton, FL: CRC Press (1984).

[34] Geurts P Ernst D Wehenkel L. Extremely randomized trees. Mach Learn. (2006) 63:3–42. 10.1007/s10994-006-6226-1

[35] Chen T Guestrin C. Xgboost: a scalable tree boosting system. In: Proceedings of the 22nd ACM SIGKDD international conference on knowledge discovery and data mining. New York, NY: ACM (2016), p. 785–94. 10.1145/2939672.2939785

[36] Freund Y Schapire RE. A decision-theoretic generalization of on-line learning and an application to boosting. J Comput Syst Sci. (1997) 55:119–39. 10.1006/jcss.1997.1504

[37] Friedman JH. Greedy function approximation: a gradient boosting machine. Ann Stat. (2001) 29:1189–232. 10.1214/aos/101320345138281721

[38] Cortes C Vapnik V. Support-vector networks. Mach Learn. (1995) 20:273–97. 10.1007/BF00994018

[39] Cover T Hart P. Nearest neighbor pattern classification. IEEE Trans Inf Theory. (1967) 13:21–7. 10.1109/TIT.1967.1053964

[40] Rish I. An empirical study of the naive Bayes classifier. In: IJCAI 2001 workshop on empirical methods in artificial intelligence, Vol. 3. Atlanta, GA (2001). p. 41–6.

[41] Dorogush AV Ershov V Gulin A. CatBoost: gradient boosting with categorical features support. arXiv. [Preprint]. arXiv:1706.09516. (2017). 10.48550/arXiv.1706.09516

[42] Sokolova M Lapalme G. A systematic analysis of performance measures for classification tasks. Inf Process Manag. (2009) 45:427–37. 10.1016/j.ipm.2009.03.002

[43] Powers DMW. Evaluation: from precision, recall and F-measure to ROC, informedness, markedness & correlation. arXiv. [Preprint]. arXiv:2010.16061. (2020). 10.48550/arXiv.2010.16061

[44] Davis J Goadrich M. The relationship between precision-recall and ROC curves. In: Proceedings of the 23rd international conference on Machine learning. New York, NY: ACM (2006), p. 233–40. 10.1145/1143844.1143874

[45] Van Rijsbergen CJ. Information Retrieval. Oxford: Butterworth-Heinemann (1979).

[46] Fawcett T. An introduction to ROC analysis. Pattern Recognit Lett. (2006) 27:861–74. 10.1016/j.patrec.2005.10.010

[47] Cohen J. A coefficient of agreement for nominal scales. Educ Psychol Meas. (1960) 20:37–46. 10.1177/001316446002000104

[48] Chicco D Jurman G. The advantages of the Matthews correlation coefficient (MCC) over F1 score and accuracy in binary classification evaluation. BMC Genomics. (2020) 21:6. 10.1186/s12864-019-6413-7 31898477 PMC6941312

[49] Lombardi A Diacono D Amoroso N Monaco A Tavares JMR Bellotti R . Explainable deep learning for personalized age prediction with brain morphology. Front Neurosci. (2021) 15:674055. 10.3389/fnins.2021.674055 34122000 PMC8192966

[50] Lucas PJ Abu-Hanna A. Prognostic methods in medicine. Citeseer. (1999). 10.1016/S0933-3657(98)00047-5 10082176

[51] Croft P Altman DG Deeks JJ Dunn KM Hay AD Hemingway H . The science of clinical practice: disease diagnosis or patient prognosis? Evidence about “what is likely to happen” should shape clinical practice. BMC Med. (2015) 13:1–8. 10.1186/s12916-014-0265-4 25637245 PMC4311412

